# Glycine Betaine-Induced Metabolic Responses Under Heat and Cold Stress in *Passiflora edulis* f. *flavicarpa*

**DOI:** 10.3390/ijms27093811

**Published:** 2026-04-24

**Authors:** Leonardo de Almeida Oliveira, Nga Thi Thu Nguyen, Darel Kenth Solde Antesco, Maryam Dabirimirhosseinlo, Naoki Terada, Atsushi Sanada, Kaihei Koshio

**Affiliations:** 1 Department of International Agricultural Development, Tokyo University of Agriculture, 1-1-1 Sakuragaoka, Setagaya City 156-0054, Tokyo, Japan; leonardoaaloliveira@gmail.com (L.d.A.O.); ngacntp@gmail.com (N.T.T.N.); dsantesco@up.edu.ph (D.K.S.A.); 13423403@nodai.ac.jp (M.D.); nt204361@nodai.ac.jp (N.T.); a3sanada@nodai.ac.jp (A.S.); 2 Faculty of Food Science and Technology, Vietnam National University of Agriculture, Trau Quy, Gia Lam, Hanoi 12406, Vietnam; 3 Institute of Crop Science, University of Philippines Los Baños, Los Baños 4031, Philippines

**Keywords:** glycine betaine, temperature stress, metabolomics, passion fruit, abiotic stress

## Abstract

Temperature extremes represent a major constraint for the cultivation of yellow passion fruit (*Passiflora edulis* Sims f. *flavicarpa*), a tropical crop increasingly exposed to heat waves and chilling events under climate change. Glycine betaine (GB) is a widely studied osmoprotectant in plants, yet its influence on metabolic responses of passion fruit under contrasting temperature stresses remains poorly characterized. This study investigated the effects of exogenous GB on primary metabolite profiles of passion fruit seedlings subjected to heat (25, 35, and 45 °C) and cold (25, 15, and 5 °C) conditions. Seedlings were treated with GB (100 mM) or left untreated, and leaf metabolites were quantified using GC–MS-based metabolomics. Heat exposure was associated with pronounced changes in amino acids, organic acids, sugars, polyamines, and γ-aminobutyric acid (GABA), while GB-treated plants showed altered levels of proline, GABA, polyamines, and selected tricarboxylic acid intermediates. Under cold conditions, several amino acids and organic acids decreased, whereas soluble sugars accumulated, particularly in GB-treated plants. Principal component analysis revealed distinct metabolic configurations under heat and cold treatments and indicated that GB modified metabolite profiles in a stress-dependent manner rather than restoring control-like states. These findings describe how GB is associated with shifts in central carbon and nitrogen metabolism under contrasting temperature regimes, providing a metabolomic perspective on stress-related metabolic adjustments in passion fruit.

## 1. Introduction

Passion fruit (*Passiflora edulis* Sims), particularly the yellow form *P. edulis* f. *flavicarpa*, is an economically important tropical and subtropical fruit crop cultivated in Latin America, Asia, and Africa, with expanding production in regions such as Brazil, China, and Japan due to its high-value juice industry and fresh-fruit markets. Climate change–driven shifts in temperature patterns are increasingly exposing passion fruit to episodes of heat and cold beyond its optimal range, threatening productivity and regional adaptation [1]. Recent work in Japan, for example, highlights passion fruit as one of the major tropical fruits grown under warm, humid conditions, but also notes increasing concern over heat stress in local production systems [2].

Temperature is a key environmental factor controlling growth, flowering, and fruit set in Passiflora. Classic experiments showed that vegetative growth and nutrient uptake in passion fruit hybrids are optimal within moderate day/night temperature regimes, while both low and high temperatures reduce growth and shift assimilate partitioning [3]. Field and pot studies further revealed that water or temperature stress during reproductive stages reduces flower number and increases bud and flower abscission, underscoring the sensitivity of passion fruit reproductive development to environmental stress [4]. More recent physiological and omics studies confirm that high summer temperatures restrict growth, shorten flowering periods, and lower fruit set in *P. edulis*, with associated changes in gene expression, photosynthesis, and hormonal signaling [5].

Beyond heat, other abiotic stresses have been used as models to understand passion fruit stress physiology. Progressive drought stress in *P. edulis* f. *edulis* leads to reduced leaf area, height and chlorophyll content, together with stomatal closure, accumulation of sugars and proline, and transcriptional activation of dehydration-responsive genes, indicating a coordinated physiological and metabolic adjustment [6]. PEG-induced water deficit and field drought experiments in *P. edulis* and triploid passion fruit further show altered photosynthetic performance, antioxidant responses, and osmolyte accumulation under stress [7]. Collectively, these studies highlight that passion fruit can adjust some components of its physiology to water and temperature stress but also confirm that its tolerance margins are relatively narrow.

At a broader scale, climate change is increasing the frequency of combined and sequential stresses: heat waves, cold snaps, and drought episodes, which impose complex, often non-additive effects on plant performance. Reviews on plant responses to climate change emphasize that multiple stress combinations strongly affect growth, photosynthesis, and yield, and that metabolite profiles under combined stress differ from those under single stresses [8]. These climatic instabilities are particularly problematic for perennial fruit crops such as passion fruit, which must maintain functional photosynthetic canopies and reproductive structures across multiple seasons.

Temperature stress, whether heat or cold, disrupts plant performance at cellular and whole-plant levels. Heat stress impairs photosystem stability, accelerates respiration, causes membrane leakage, and enhances oxidative damage, while cold stress reduces membrane fluidity, slows enzymatic reactions, and limits carbon assimilation [9]. In both cases, plants rely on defense mechanisms such as heat-shock proteins, changes in membrane lipid composition, and enhanced antioxidant systems to re-establish homeostasis. However, many tropical crops and fruit species are inherently less tolerant to chilling and extreme heat compared with temperate species, making their seedling stages especially vulnerable.

Central to plant adjustment to temperature and other abiotic stresses is metabolic response. Omics-based studies have shown that central carbon metabolism, amino acid biosynthesis, the tricarboxylic acid (TCA) cycle, and associated organic acids are extensively remodeled under drought, salinity, heat, cold, and their combinations [10]. Accumulation of compatible solutes (e.g., proline and other amino acids), soluble sugars, sugar alcohols, and specific organic acids is a hallmark of plant responses, helping to maintain osmotic balance, protect macromolecules, buffer pH, and support energy metabolism under stress [11]. Although such metabolite shifts have been characterized in several model and crop species, information on temperature-driven metabolic adjustments in *Passiflora edulis*, particularly during seedling stages, is still scarce.

Among the compatible solutes involved in abiotic stress tolerance, glycine betaine (GB) has attracted long-standing interest. It is a quaternary ammonium compound that accumulates in chloroplasts and other cellular compartments of many natural GB accumulators, where it stabilizes proteins and membranes, protects the photosynthetic apparatus, and contributes to ROS detoxification [12]. Classic and recent reviews consistently show that GB improves tolerance to drought, salinity, and temperature extremes in numerous species by maintaining osmotic adjustment, preserving photosystem II function, and modulating antioxidant and hormonal signaling [13].

GB can be increased in plants either by exogenous application or by engineering its biosynthetic pathway. Transgenic approaches introducing genes for GB synthesis have enhanced tolerance to various abiotic stresses in otherwise weak accumulators, although accumulated levels are sometimes lower than in natural GB-accumulating species [14]. Meanwhile, foliar or soil application of GB has been widely reported to improve plant water status, chlorophyll retention, and yield components under stress in crops such as wheat, maize, rice, and tomato [12]. Recent syntheses also emphasize GB’s role in fine-tuning stress hormone pathways, ion homeostasis, and redox balance, reinforcing its potential as a practical tool for climate-smart crop management [13].

Despite this extensive knowledge in other crops, there is almost no information on how GB influences the metabolism of *Passiflora edulis* under temperature stress. Existing passion fruit studies under drought and high temperature have focused mainly on morphology, water relations, photosynthetic parameters, transcriptomics, and phosphoproteomics, revealing stress-induced changes in sugar metabolism, osmolyte levels, and regulatory proteins [6], they rarely provide a comprehensive view of temperature-dependent shifts in amino acids, organic acids, and sugars within a controlled thermal gradient, and none specifically investigate GB as a metabolic modulator in passion fruit seedlings.

Furthermore, many plant stress studies treat heat and cold independently, even though these stresses impose contrasting but sometimes overlapping challenges to cellular metabolism. Comparative metabolomic work in other crops shows that heat and cold can generate distinct “metabolic fingerprints,” with differential accumulation of amino acids, sugars, flavonoids, and organic acids linked to cultivar-specific tolerance [15]. Yet, similar comparative analyses are lacking for *P. edulis*, especially in the early seedling phase where thermal limits for growth and metabolic integrity are still being defined.

Despite increasing research on the role of glycine betaine in plant stress physiology, its influence on primary metabolic responses of *Passiflora edulis* under contrasting temperature stresses remains poorly characterized. Understanding these metabolic adjustments is important for interpreting how tropical crops respond to both heat and chilling conditions. Therefore, the objective of this study was to characterize changes in primary metabolite profiles of *Passiflora edulis* seedlings exposed to heat and cold stress with or without exogenous glycine betaine. We hypothesized that GB treatment would be associated with distinct metabolic adjustments under contrasting temperature regimes rather than simply restoring metabolite levels to control conditions. To test this hypothesis, GC–MS–based metabolomic profiling was used to quantify primary metabolites in leaves of passion fruit seedlings subjected to controlled temperature treatments.

## 2. Results

According to Table 1, proline content was significantly influenced by both temperature treatment and glycine betaine (GB) application. Under control conditions (H0), GB-treated seedlings (H0GB1) exhibited substantially higher proline levels compared with untreated plants (H0GB0). A similar pattern was observed under mild heat stress (H1), where GB application was associated with increased proline accumulation relative to the corresponding control treatment. Under severe heat stress (H2), proline levels remained low in untreated seedlings but increased markedly in GB-treated plants, reaching the highest values observed in the experiment.

Several nitrogen-related metabolites, including proline, responded to both temperature treatment and glycine betaine (GB) application. Proline concentrations increased markedly with GB application across temperature conditions, particularly under severe heat stress, where GB-treated seedlings exhibited the highest proline levels observed in the experiment. γ-Aminobutyric acid (GABA) showed a similar pattern: concentrations remained comparable between GB treatments under control temperature but increased substantially with GB application under both mild and severe heat stress. Glutamic acid and glutamine also exhibited higher levels in GB-treated plants, with the strongest increases observed under mild heat stress conditions. Valine concentrations were generally low but increased with GB application under control and severe heat conditions.

Metabolites associated with the polyamine pathway also showed clear treatment responses. Putrescine concentrations were consistently higher in GB-treated plants compared with untreated controls, with the most pronounced accumulation occurring under mild heat stress. Ornithine exhibited substantial variation among treatments and increased markedly under mild heat stress combined with GB application, while remaining comparatively low under severe heat stress.

Carbohydrate and organic acid metabolism also responded to heat and GB treatments. Sucrose accumulated strongly under mild heat stress regardless of GB treatment, whereas concentrations remained relatively low under control and severe heat conditions. Glucose concentrations showed distinct variation across treatments: under mild heat stress, glucose levels declined in untreated plants but increased substantially in GB-treated seedlings. Malic acid showed pronounced increases under mild heat stress, particularly in GB-treated plants, while concentrations remained lower under control and severe heat conditions.

Metabolic responses under cold stress are summarized in Table 2. Proline concentrations differed markedly across temperature treatments. Under control temperature (C0), proline levels remained high and similar between GB-treated and untreated plants. However, under mild cold stress (C1), proline concentrations decreased sharply in both treatments. Under severe cold stress (C2), proline levels remained low overall, although GB-treated seedlings exhibited slightly higher concentrations than untreated plants.

Sucrose concentrations increased under cold stress, particularly with GB application. While sucrose remained low and similar between GB treatments under control temperature (C0), levels increased under mild cold stress (C1) and were consistently higher in GB-treated seedlings. Under severe cold stress (C2), sucrose remained moderate in untreated plants but increased further with GB application.

Glucose also increased under cold stress conditions. Although concentrations remained extremely low under control temperature (C0), glucose increased under mild cold stress (C1) and remained relatively high under severe cold stress (C2), with limited differences between GB treatments at the lowest temperature.

In contrast, fructose and sorbitol showed strong declines under cold stress. Both metabolites exhibited high concentrations under control temperature but decreased drastically under mild and severe cold conditions, with no clear recovery associated with GB treatment.

Inositol showed high concentrations under control temperature and decreased under mild cold stress. Under severe cold stress, inositol levels increased in untreated plants but declined again in GB-treated seedlings.

Organic acids also responded strongly to cold stress. Malic acid showed very high concentrations under control temperature but decreased markedly under both cold stress levels. However, GB-treated seedlings consistently exhibited higher malic acid concentrations than untreated plants under both C1 and C2 conditions. Citric acid followed a similar pattern, with the highest concentrations observed under control temperature and substantially lower levels under cold stress.

Among amino acids, threonine showed high concentrations under control temperature but decreased sharply under both cold stress treatments. Putrescine also declined under cold stress, with relatively high values under C0 and substantially lower concentrations under C1 and C2 in both GB treatments.

**Figure 1:**
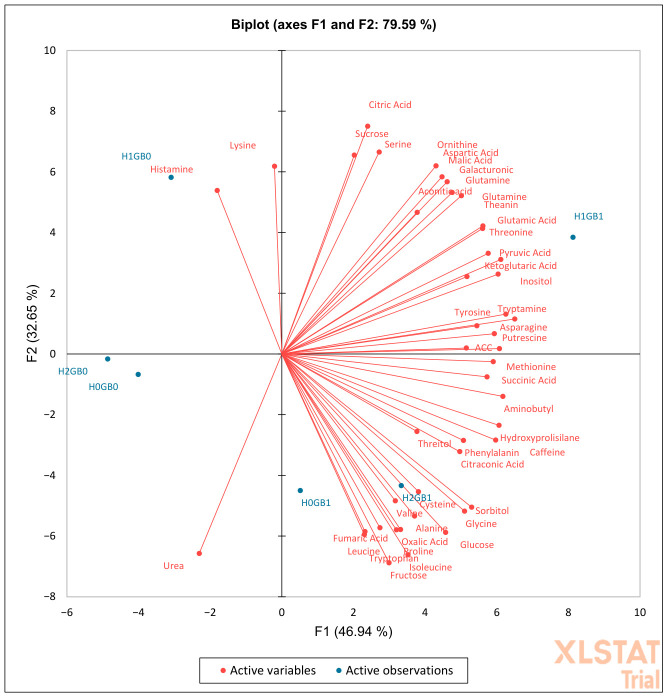
Principal Component Analysis (PCA) of passion fruit plants submitted to heat stress. “H0” refers to control heat, “H1” indicates mild heat, and “H2” denotes severe heat. “GB0” indicates no application of glycine betaine, whereas “GB1” signifies glycine betaine application.

Principal component analysis (PCA) was conducted to explore overall metabolic patterns associated with heat stress and glycine betaine (GB) application. The first two principal components explained 79.59% of the total variance in the dataset (PC1 = 46.94%, PC2 = 32.65%) (Figure 1). The score plot indicated separation of treatments primarily along PC1, with H0GB0 and H2GB0 located on the negative side of PC1, whereas H0GB1, H2GB1, and H1GB1 were positioned on the positive side. H1GB0 was separated from the other treatments mainly along PC2. The loading plot showed that numerous metabolites contributed positively to PC1, including several amino acids and organic acids such as methionine, citric acid, aconitic acid, threonine, glutamine, asparagine, aspartic acid, and α-ketoglutaric acid.

Polyamine-related compounds such as γ-aminobutyric acid (GABA) and putrescine, as well as soluble sugars including glucose and fructose, also contributed strongly to the positive direction of PC1. Positive PC2 values were associated with metabolites such as citric acid, serine, and ornithine, whereas negative PC2 values were associated with fructose, glucose, and several metabolites clustered in the lower-right region of the plot.

Overall, PCA revealed distinct metabolic patterns among heat treatments and GB applications, indicating that temperature stress and GB treatment were associated with shifts in metabolite profiles across the dataset.

**Figure 2:**
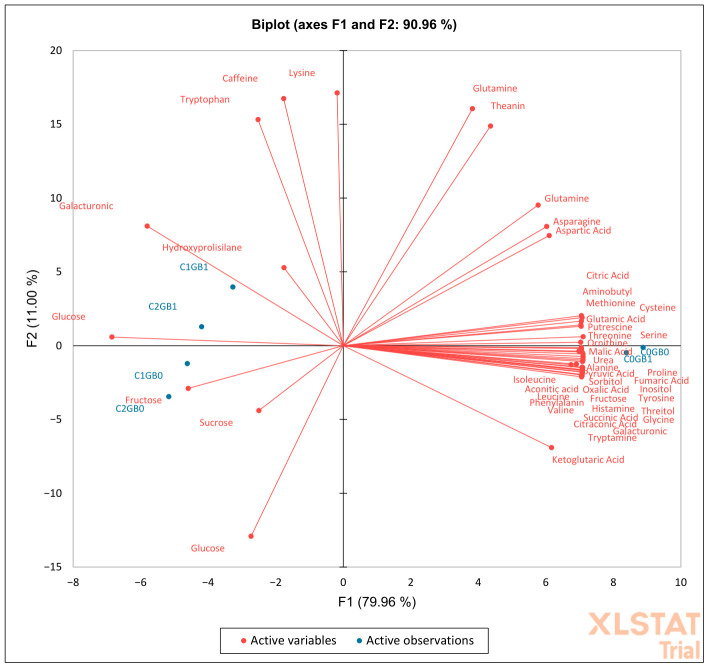
Principal Component Analysis (PCA) of the metabolites of passion fruit submitted to cold stress. “C0” refers to control cold, “C1” indicates mild cold, and “C2” denotes severe cold. “GB0” indicates no application of glycine betaine, whereas “GB1” signifies glycine betaine application.

Principal component analysis (PCA) of metabolite profiles under cold stress showed that the first two principal components explained 90.96% of the total variance, with PC1 accounting for 79.96% and PC2 accounting for 11.00% (Figure 2). PC1 primarily separated control samples from cold-stressed treatments. Samples corresponding to C0GB0 and C0GB1 were positioned on the positive side of PC1, whereas cold-stressed samples (C1GB0, C2GB0, C1GB1, and C2GB1) were shifted toward the negative side. Differences among the cold-stressed treatments were mainly distributed along PC2.

In the loading plot, positive PC1 values were associated with a cluster of metabolites dominated by organic acids and amino acids, including citric acid, aconitic acid, α-ketoglutaric acid, aspartic acid, asparagine, glutamine, ornithine, and threonine. In contrast, negative PC1 values were associated with several soluble sugars and related metabolites, including glucose, fructose, sucrose, galacturonic acid, caffeine and tryptophan. This distribution suggests that cold stress was associated with a shift in the relative contribution of metabolites, with organic acids and amino acids contributing more strongly to the control condition, while soluble sugars were more strongly associated with cold-stressed treatments.

PC2 contributed to additional separation among cold-stressed treatments. Positive PC2 values were associated with metabolites such as lysine, tryptophan, caffeine, glutamine, and theanine, whereas negative PC2 values were associated with glucose, sucrose, α-ketoglutaric acid, and fructose. Cold-stressed treatments without GB application (C1GB0 and C2GB0) were located toward the negative side of PC1 and showed greater dispersion along PC2. In contrast, GB-treated cold-stressed samples (C1GB1 and C2GB1) were positioned closer to the origin and slightly shifted toward the positive side of PC1 relative to their untreated counterparts. This pattern suggests that GB treatment was associated with a partial shift in the overall metabolite distribution under cold stress conditions.

**Figure 3:**
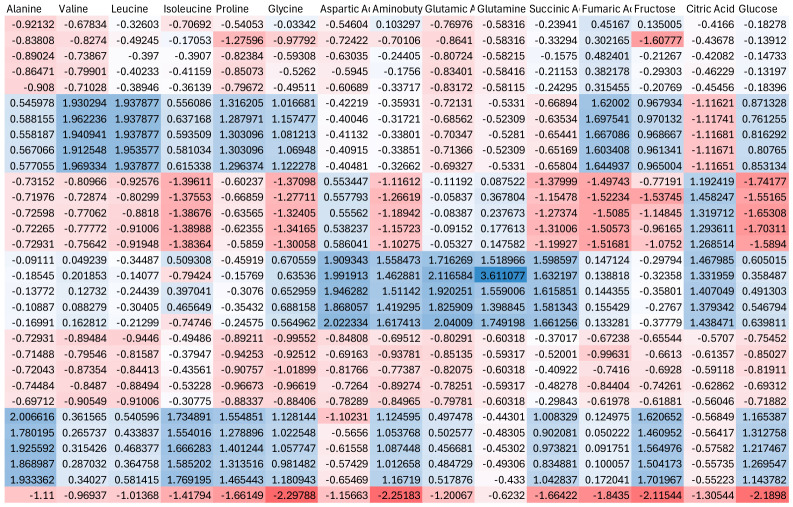
Heatmap showing Z-score normalized metabolite profiles under heat stress conditions. Red indicates higher relative abundance, while blue indicates lower relative abundance.

Heat stress induced pronounced changes in metabolite profiles, as shown by the heatmap of Z-score normalized data (Figure 3). Amino acids, including proline, valine, leucine, isoleucine, and alanine, exhibited clear increases under heat conditions, indicating enhanced nitrogen metabolism and stress-related adjustments. Additionally, organic acids such as succinic, fumaric, and citric acids displayed coordinated changes, suggesting alterations in central carbon metabolism. Sugars, including glucose and fructose, also showed variable responses, reflecting shifts in energy balance and osmotic regulation. Overall, heat stress was associated with increased metabolic activity and reorganization of primary metabolic pathways.

**Figure 4:**
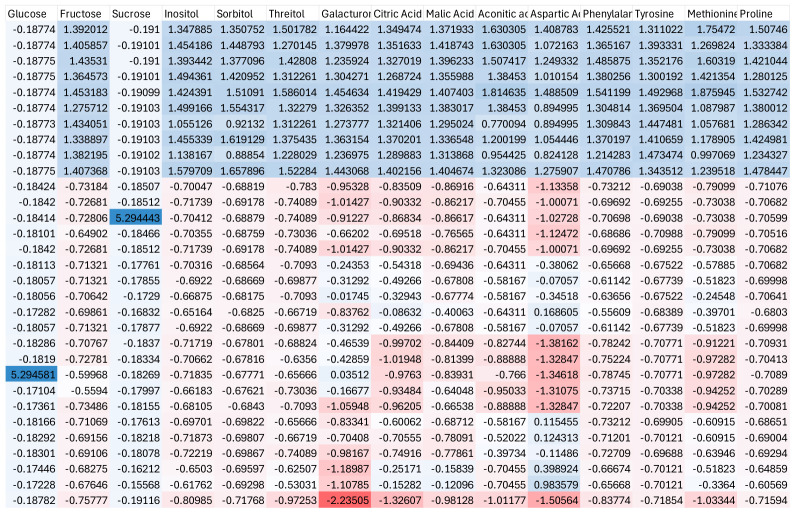
Heatmap showing Z-score normalized metabolite profiles under cold stress conditions. Red indicates higher relative abundance, while blue indicates lower relative abundance.

In contrast, cold stress resulted in a distinct metabolic pattern characterized by the accumulation of carbohydrates and selective modulation of amino acids (Figure 4). Sugars such as glucose, fructose, and sucrose showed higher relative abundance, along with sugar alcohols including inositol and sorbitol, indicating osmotic adjustment and carbon storage under low-temperature conditions. Organic acids, including malic and citric acids, exhibited variable responses, suggesting constrained metabolic flux. Amino acids showed less pronounced and more heterogeneous changes compared to heat stress. These results indicate that cold stress primarily promotes carbon accumulation and metabolic conservation rather than increased turnover.

Together, these results demonstrate that heat and cold stress induce contrasting metabolic adjustments, with heat promoting increased metabolic turnover and cold favoring carbon accumulation and conservation.

## 3. Discussion

The present study shows that exogenous glycine betaine (GB) substantially reshaped the primary metabolite profile of yellow passion fruit leaves exposed to both heat and cold stress. In general, GB application was associated with increased accumulation of compatible solutes such as proline, selected amino acids, soluble sugars and certain organic acids under stressful temperatures, while partially moderating the depletion of several metabolites that were strongly reduced by cold or heat in untreated plants. These findings are consistent with the widely reported role of GB as an osmoprotective compound and metabolic modulator that has been associated with stabilization of proteins and membranes, protection of the photosynthetic apparatus, and maintenance of cellular redox balance under diverse abiotic stresses, including salinity, drought, heat and chilling [12,13,16,17,18].

Although *Passiflora* species are not generally considered strong natural accumulators of GB, the present results suggest that exogenous GB application can still be associated with metabolomic adjustments comparable to those reported in species engineered to synthesize GB de novo or in crops receiving foliar GB treatments [14,19]. This observation supports the view that the protective effects of GB may extend beyond its direct osmotic contribution and may involve broader modulation of metabolic pathways, hormone signaling and antioxidant systems [13].

Under heat stress, GB application was associated with enhanced proline accumulation, particularly at severe temperatures where H2GB1 leaves exhibited substantially higher proline levels than the corresponding GB0 treatment. This pattern is consistent with numerous studies describing coordinated relationships between GB and proline, in which both osmolytes contribute to osmotic adjustment, ROS detoxification and stabilization of macromolecular structures under stress conditions [12,20]. Proline is increasingly recognized not only as a compatible solute but also as a component of cellular redox buffering and a signaling molecule involved in stress-responsive gene regulation and programmed cell death pathways [21]. The strong proline accumulation observed in GB-treated passion fruit under heat stress may therefore reflect metabolic adjustments that favor osmoprotection and redox balance.

Although enzyme activities were not measured in the present study, several mechanisms described in the literature may help explain how exogenous GB is associated with the metabolic patterns observed here. GB has been reported not only as an osmoprotectant but also as a compatible solute capable of influencing stress-responsive gene expression and enzyme activities in several plant species [12,13,22]. For example, GB treatment has been associated with increased expression or activity of Δ^1^-pyrroline-5-carboxylate synthase (P5CS), the key regulatory enzyme in proline biosynthesis, thereby promoting proline accumulation under abiotic stress conditions [12,20]. Similarly, polyamine biosynthesis pathways involving arginine decarboxylase (ADC) and ornithine decarboxylase (ODC) are frequently stimulated during stress responses where GB is present, contributing to increased putrescine production and related metabolites that participate in membrane stabilization and ROS signaling networks [23,24].

In addition to influencing amino acid and polyamine metabolism, GB has been widely reported to contribute to stabilization of membrane structures and protection of photosystem II under thermal stress, which may help limit excessive reactive oxygen species (ROS) generation [16,22]. Reduced oxidative damage may in turn help preserve enzyme integrity within central carbon metabolism, indirectly supporting tricarboxylic acid (TCA) cycle intermediates and associated nitrogen assimilation pathways. Therefore, the metabolite patterns observed in this study are consistent with a model in which GB is associated with structural stabilization and modulation of stress-responsive metabolic networks, promoting coordinated changes in proline, polyamines, organic acids and soluble sugars under temperature stress.

Heat stress also induced marked changes in glutamic acid, glutamine and γ-aminobutyric acid (GABA), particularly in the presence of GB. Glutamate occupies a central position at the intersection of nitrogen assimilation and the TCA cycle, serving as a precursor for both proline and GABA biosynthesis [25]. The increase in glutamate and glutamine under mild heat stress with GB treatment may reflect enhanced nitrogen remobilization and reassimilation processes that support the synthesis of stress-related metabolites. GABA accumulation has been widely reported in plants subjected to heat, hypoxia or mechanical stress and is associated with activation of the GABA shunt, an alternative pathway that bypasses segments of the TCA cycle while contributing to carbon–nitrogen balance and intracellular pH regulation [25]. The marked increase in GABA observed in GB-treated leaves under H1 and H2 therefore suggests that GB may be associated with activation of this alternative metabolic route, potentially contributing to metabolic flexibility during heat stress.

Polyamine metabolism was also responsive to the combined effects of GB application and temperature stress. Putrescine and its precursor ornithine increased substantially in GB-treated leaves under mild and severe heat conditions. Polyamines are widely recognized for their roles in stabilizing membranes and nucleic acids, modulating ion channels, and interacting with ROS signaling pathways during plant stress responses [10]. The higher putrescine concentrations observed in GB-treated plants may therefore reflect metabolic adjustments associated with a more protective polyamine profile under elevated temperatures. This interpretation is consistent with previous reports indicating that GB treatments or GB overaccumulation can be associated with increased polyamine levels in stressed plants and that polyamine oxidases interact with GABA metabolism and ROS homeostasis during both cold and heat adjustment [10,23].

The most pronounced effect of heat within the carbohydrate fraction was the substantial increase in sucrose and glucose under mild heat stress, particularly in GB-treated plants. Heat stress is known to enhance respiratory activity and alter source–sink relationships, which can lead to soluble sugar accumulation either through impaired phloem export or through increased starch degradation [10,25]. Several metabolomic studies have reported that sucrose, glucose, and fructose often accumulate under high temperature conditions and may function both as compatible solutes and as signaling molecules involved in regulating stress-responsive gene expression and growth recovery [10,25]. The relatively higher sucrose and glucose levels observed in GB-treated passion fruit under H1 and H2 compared with GB0 plants may therefore indicate that GB application was associated with a more stable carbohydrate pool under heat stress.

Malic acid also showed a strong response to heat, with elevated levels under mild heat conditions in GB-treated leaves. Organic acids of the tricarboxylic acid (TCA) cycle, such as malate and citrate, play key roles in respiratory energy production, anaplerotic carbon supply and redox balancing between cellular compartments [10,25]. The higher malate concentrations observed under GB treatment may reflect adjustments in central carbon metabolism that support respiratory activity under heat stress. These observations are consistent with previous studies suggesting that GB treatment can contribute to stabilization of cellular structures and metabolic processes during thermal stress, potentially supporting the continued operation of respiration-related pathways [12,16].

Overall, the metabolite profile observed under heat stress in GB-treated passion fruit is consistent with the broader view that extreme temperatures induce substantial responses of plant central metabolism, and that compatible solutes such as GB may be associated with metabolic states characterized by enhanced osmotic buffering, redox balance and energy metabolism adjustments [10,25]. These patterns are consistent with well-established metabolic responses of plants to temperature stress.

In contrast to the heat experiment, the cold treatment produced a different pattern for several metabolites. Cold stress is commonly associated with shifts toward carbohydrate accumulation and metabolic conservation, reflecting adaptive responses to low-temperature conditions. Proline levels were already relatively high under the control cold temperature and decreased sharply under both mild and severe cold conditions, with only partial recovery when GB was applied at 5 °C. This pattern differs from the classical response described in many temperate plant species, where cold acclimation is often accompanied by increased proline accumulation [26]. Several factors may explain this difference. First, tropical species such as passion fruit may downregulate proline biosynthesis under very low temperatures to limit metabolic energy costs. Second, proline may be rapidly oxidized to support mitochondrial respiration when other carbon substrates are limited. Third, the relatively short daily cold exposure applied in this experiment may not have been sufficient to induce a typical cold-adjustment response. Nevertheless, the slightly higher proline levels observed in GB-treated plants at the most severe cold temperature suggest that GB application may partially mitigate the reduction in proline metabolism under strong chilling conditions.

The marked decline in proline under cold stress contrasts with the classical response observed in many temperate species, where cold adjustment is frequently accompanied by proline accumulation [26]. Proline biosynthesis from glutamate requires ATP and reducing equivalents through the P5CS–P5CR pathway, representing a metabolically demanding process [20,21]. Under low temperatures, reduced enzymatic activity and constrained mitochondrial respiration may limit the availability of energy and reducing power, potentially leading to suppression of energetically expensive biosynthetic pathways. In addition, proline can be oxidized via proline dehydrogenase (ProDH) and reintroduced into central carbon metabolism, contributing carbon skeletons to the TCA cycle and supporting respiratory metabolism under stress conditions [20,21]. Therefore, the decrease in proline observed under chilling conditions may reflect either reduced biosynthesis or enhanced catabolic utilization as an alternative respiratory substrate. This interpretation is consistent with the concurrent reduction in organic acids and the accumulation of soluble sugars observed in the PCA, suggesting a broader reorganization of central metabolism and altered carbon partitioning during cold stress [10,25,27].

In contrast, soluble sugars exhibited a more typical cold-response pattern. Sucrose, glucose and fructose increased markedly under both mild and severe cold conditions, particularly in GB-treated leaves. Accumulation of sucrose and hexoses during cold exposure has been widely reported and is considered an important component of cold adjustment responses because these sugars can stabilize membranes and proteins, influence the physical properties of cellular solutions and participate in the regulation of cold-responsive gene expression pathways such as the CBF regulatory network [24,26,28]. The stronger sugar accumulation observed in GB-treated plants may therefore indicate that GB application was associated with greater retention or redistribution of soluble carbohydrates under chilling conditions.

Interestingly, polyols such as sorbitol and inositol followed a contrasting pattern, showing relatively high levels under control conditions but decreasing sharply under cold stress, with limited recovery in GB-treated plants. Polyols often function as compatible solutes and ROS scavengers during drought and salinity stress, although their responses to cold stress appear to vary considerably among plant species and tissues [17,18]. In passion fruit, the decline of sorbitol and inositol under C1 and C2 conditions may indicate conversion into other metabolic intermediates or redistribution to other tissues. The limited influence of GB on these compounds suggests that the metabolic adjustments associated with GB treatment in this species may rely more strongly on soluble sugar and organic acid regulation than on polyol accumulation.

Malic and citric acids were abundant under control conditions but declined markedly under cold stress, with GB treatment partially mitigating these decreases, particularly at the most severe cold temperature. This pattern may reflect a reduction in TCA-cycle activity and/or a shift in carbon allocation away from organic acid pools toward sugars and other soluble metabolites. Similar decreases in TCA intermediates during cold exposure have been reported in *Arabidopsis* and other species, where respiration is often downregulated during cold adjustment and specific organic acids can be redirected toward amino acid or secondary metabolite biosynthesis [10,25]. The slightly higher malate and citrate levels observed in GB-treated plants under severe cold may therefore indicate that GB application was associated with partial maintenance of organic acid pools during chilling stress.

Threonine and putrescine both declined strongly under cold stress in GB0 plants, reflecting changes in nitrogen metabolism under low temperature conditions. Threonine often accumulates under stress as a compatible solute and as a precursor for isoleucine and other downstream metabolites, although its response is highly species-dependent [25,29]. The pronounced decrease observed here suggests that passion fruit may prioritize other amino acids, such as glutamate and GABA, under chilling conditions. Putrescine, in turn, is a central polyamine whose accumulation is frequently associated with cold stress responses and ROS regulation [24,30]. Its reduction in cold-stressed GB0 plants may therefore indicate decreased availability of polyamine-related protective metabolites. The slightly higher putrescine levels observed in GB1 leaves under cold conditions, although still lower than under control temperature, suggest that GB treatment was associated with partial maintenance of the polyamine pool, which may complement the increased soluble sugar levels observed under chilling.

The heat-stress PCA showed that multiple amino acids, organic acids, and soluble sugars loaded in similar directions along the dominant axis, a pattern frequently reported in heat-stress metabolomics studies and interpreted as evidence of coordinated changes in central metabolism, including glycolysis, pyruvate entry points, the tricarboxylic acid (TCA) cycle, and amino acid biosynthesis [10]. In particular, nitrogen hub metabolites such as glutamate, glutamine, and aspartate clustered together with TCA intermediates including citric, malic, and aconitic acids, as well as polyamine-related compounds such as putrescine and γ-aminobutyric acid (GABA). This coordinated loading pattern suggests close coupling between nitrogen assimilation and carbon metabolism under heat stress. Such integration is consistent with increased metabolic turnover, where respiratory activity and amino acid interconversion may contribute to ATP production, osmotic adjustment, and redox balance during thermal stress. Although PCA does not directly quantify metabolic fluxes, the clustering of these metabolites supports the interpretation of coordinated metabolic adjustments in heat-exposed plants, particularly under GB treatment.

Respiratory metabolism is particularly sensitive to elevated temperatures. High leaf temperatures can increase respiratory demand while simultaneously affecting enzyme stability, leading to altered pools of organic acids and nitrogen-containing metabolites that feed back into overall metabolic balance [31]. Consistent with this framework, heat stress across plant species is often associated with increased abundance of TCA intermediates and amino acids, reflecting intensified metabolic turnover rather than simple metabolic inhibition.

Amino acid accumulation under heat stress is widely reported and reflects their multifunctional roles as nitrogen storage compounds, intermediates of carbon metabolism, redox buffers, and participants in stress signaling pathways [32]. Organic acids and soluble sugars frequently change in parallel, reinforcing the concept that heat stress triggers system-level metabolic responses rather than isolated metabolite alterations.

In *Passiflora edulis*, heat sensitivity has been documented particularly during reproductive development, with high temperatures shown to shorten flowering periods, increase bud abscission, and reduce fruit set [5]. Molecular studies further demonstrate that heat stress disrupts transcriptional and post-translational regulation in passion fruit tissues, supporting the relevance of the metabolic separation observed in the PCA under heat stress.

Glycine betaine (GB) is widely recognized as a compatible solute that has been associated with stabilization of proteins and membranes and with maintenance of physiological processes under heat stress conditions [22]. Accordingly, the distinct positioning of GB-treated heat-stressed samples in the PCA space suggests that GB application was associated with a modified metabolic configuration rather than a return to control-like profiles, consistent with a role of GB as a modulator of stress-related metabolic adjustments.

Cold stress and chilling exposure are characterized by strong and highly coordinated metabolic responses, often resulting in dominant principal components in multivariate analyses [27]. In the present study, the cold PCA captured a large proportion of variance in the first component, consistent with cold acting as a major organizing factor of the metabolic state.

A central feature of cold responses is the reorganization of carbohydrate metabolism. Low temperatures reduce enzymatic reaction rates and growth demand, frequently leading to the accumulation of soluble sugars and related carbohydrates as carbon utilization slows [27]. These carbohydrates function not only as osmoprotectants but also as metabolic buffers and signaling molecules during cold exposure. In the present study, the PCA clearly separated cold-stressed samples along the dominant axis, with soluble sugars such as glucose, fructose, and sucrose loading opposite to several organic acids and amino acids. This contrasting distribution suggests a shift in metabolic emphasis from organic-acid and amino-acid pools toward soluble carbohydrate accumulation under chilling conditions. The reduced association of TCA intermediates such as malate and citrate with cold-treated plants further supports the interpretation of altered respiratory metabolism and modified carbon partitioning at low temperature. Although PCA does not directly measure metabolic fluxes, the strong loading of sugars along the cold-associated axis is consistent with carbon reallocation toward carbohydrate pools during chilling stress [27].

Cold adjustment is regulated by well-characterized molecular networks, including the CBF/DREB signaling pathway, which coordinates transcriptional, physiological, and metabolic responses to low temperature [33]. Although the present analysis focuses on metabolite profiles rather than gene expression, the structured separation observed in the PCA is consistent with the operation of integrated regulatory responses to cold stress.

Chilling sensitivity is a recognized limitation in *Passiflora edulis*. Comparative transcriptomic studies have identified distinct molecular responses between chilling-tolerant and chilling-sensitive passion fruit genotypes, confirming that cold stress represents a significant constraint for this crop [34]. These findings provide species-specific context for the pronounced metabolic separation observed under cold stress in the present study.

Glycine betaine (GB) has been widely reported to contribute to cold tolerance by stabilizing cellular structures and supporting metabolic function under low temperature conditions [35]. The positioning of GB-treated cold-stressed samples closer to control samples in multivariate space is therefore consistent with previously described roles of GB in modulating stress-related metabolic responses under chilling conditions, while still reflecting the persistence of cold-induced metabolic adjustments.

Although heat and cold represent opposite thermal extremes, both impose substantial constraints on cellular homeostasis and trigger broad metabolic responses. A common feature across temperature stresses is the involvement of reactive oxygen species (ROS) and redox signaling as central components of stress perception and downstream metabolic responses [36].

Despite this shared redox component, heat and cold produce distinct metabolic architectures. Heat stress is frequently associated with increased metabolic turnover involving amino acids, organic acids, and sugars, reflecting intensified activity within central metabolic pathways [10]. In contrast, cold stress is characterized by dominant reorganization of carbohydrate pools and reduced metabolic throughput, consistent with kinetic limitations and altered carbon partitioning at low temperatures [27].

Across both temperature stresses, GB application was associated with systematic shifts in multivariate metabolite space. Rather than eliminating stress responses, GB appears to be associated with stress-specific metabolic configurations that differ between heat and cold conditions. This pattern aligns with extensive literature describing GB as a modulator of abiotic stress responses through stabilization of macromolecules, maintenance of metabolic connectivity, and support of physiological processes [12,22].

Importantly, the PCA patterns indicate that GB did not simply restore stressed plants to a control-like metabolic state. Under heat stress, GB-treated samples occupied a distinct region of multivariate space rather than overlapping with control treatments, reflecting coordinated changes involving amino acids, TCA intermediates, polyamines, and soluble sugars. Similarly, under cold stress, GB-treated samples remained clearly separated from control plants along the dominant principal component, despite partial displacement toward the control region. These configurations suggest that GB is associated with modifications of the metabolic architecture of stressed plants rather than reversing stress-induced changes. At a systems level, PCA captures coordinated covariance among metabolites, and the clustering patterns observed here are consistent with GB being associated with stress-specific metabolic adjustments that differ between heat and cold conditions rather than a complete restoration of pre-stress metabolic states.

Recent reviews emphasize that metabolite profiles integrate multiple layers of stress response and can serve as indicators of metabolic organization under adverse environmental conditions [8]. The contrasting yet coherent PCA patterns observed under heat and cold stress therefore support the use of metabolomics as an approach to explore temperature-specific stress responses and to examine the role of metabolic modulators such as glycine betaine in perennial fruit crops.

Our observations are broadly consistent with earlier studies reporting that exogenous GB application is associated with improved performance of horticultural crops under heat and cold stress, often accompanied by changes in osmolyte accumulation, photosynthetic performance and membrane stability. GB treatments have been reported to improve heat tolerance and yield in tomato, marigold and apple, and to enhance chilling tolerance in tomato, cucumber, maize, chickpea and pomegranate fruits [37,38,39,40,41,42,43]. In many of these studies, GB-treated plants exhibited higher proline and sugar contents, improved maintenance of PSII efficiency and reduced indicators of oxidative damage under stress conditions, suggesting that GB may contribute to both osmotic adjustment and antioxidant protection [12,16,44,45].

From a metabolomics perspective, the combined shifts in amino acids, sugars and organic acids observed in GB-treated passion fruit are consistent with the framework proposed by [25] and [10], who emphasized that abiotic stresses induce coordinated adjustments of central metabolism to maintain energy balance, redox status and osmotic stability. Proline, GABA, TCA intermediates and soluble sugars represent key metabolic nodes within this network. Their differential responses to heat and cold in the present study highlight that, even when the same compound (GB) is applied, the resulting metabolic configuration appears to be stress-specific: under heat, GB was associated with increased proline, GABA, polyamines and malate, whereas under cold conditions GB treatment corresponded with greater accumulation of soluble sugars and partial maintenance of organic acids and polyamines.

The heatmap analysis further highlights the contrasting metabolic responses induced by heat and cold stress, reinforcing the distinct patterns observed in the multivariate analysis. Under heat stress, the coordinated accumulation of several amino acids, including proline, branched-chain amino acids (valine, leucine, and isoleucine), and alanine, suggests an overall increase in nitrogen metabolism and protein turnover. This response is commonly associated with stress adaptation mechanisms, where amino acids function as osmoprotectants, alternative energy sources, and signaling molecules. In parallel, the modulation of tricarboxylic acid (TCA) cycle intermediates, such as succinic, fumaric, and citric acids, indicates enhanced metabolic flux and energy demand under elevated temperatures.

In contrast, cold stress resulted in a metabolic configuration dominated by carbohydrate accumulation, as evidenced by the increased relative abundance of glucose, fructose, sucrose, and sugar alcohols such as inositol and sorbitol. This pattern reflects a shift toward carbon storage and osmotic adjustment, which are critical for maintaining cellular integrity under low-temperature conditions. The comparatively moderate and heterogeneous changes observed in amino acids further support the idea that cold stress imposes constraints on metabolic activity, limiting extensive metabolic reprogramming.

Although glycine betaine application did not eliminate stress-induced metabolic responses, it contributed to reshaping metabolite profiles under both temperature extremes, suggesting a modulatory role in metabolic adjustment rather than a complete restoration to control conditions.

Yellow passion fruit is highly valued in tropical and subtropical regions but remains vulnerable to temperature extremes, particularly the combined occurrence of heat waves and cold spells associated with climate variability. The present results indicate that exogenous GB application can substantially influence leaf metabolite profiles under both high and low temperature conditions. In practical terms, GB-treated plants showed higher levels of several compatible solutes such as proline and sugars, partial maintenance of TCA intermediates under severe cold, and increased abundance of GABA and polyamines under heat conditions. These metabolic patterns are consistent with responses previously associated with improved stress performance in other crop species [10,12,16,23,25].

Nevertheless, the contrasting behaviour of several metabolites—particularly the decrease in proline and polyols under cold—suggests that passion fruit, as a tropical species, may rely on stress strategies that differ from those described in temperate model plants commonly used in cold adjustment research [24,26,28]. Future studies should therefore combine the metabolite profiles reported here with measurements of physiological traits (e.g., chlorophyll fluorescence, gas exchange, membrane leakage and ROS markers) as well as transcriptomic analyses of key genes involved in proline, GB, GABA and polyamine metabolism. Integrating these datasets would help clarify whether GB primarily acts through stabilization of metabolic processes or through broader regulation of gene expression in passion fruit, as has been reported in other species [10,13,45,46].

Another limitation of the present study is that metabolite measurements were obtained at a single time point following stress exposure. Previous studies indicate that metabolite responses to temperature can be highly dynamic, with transient peaks followed by stabilization or decline during adjustment [10,25]. Time-course experiments, including recovery phases after re-warming or return to optimal temperature, would therefore help distinguish acute stress responses from longer-term adjustment processes. In addition, extending the analysis to reproductive tissues and fruits will be important to determine whether the GB-associated metabolic adjustments observed in leaves translate into improved flowering, fruit set and juice quality—traits of direct agronomic relevance for passion fruit production.

In this study, temperature treatments were applied using independent growth chambers. Therefore, temperature was effectively imposed at the chamber level rather than the individual plant level. Although all chambers were maintained under identical environmental conditions aside from temperature, chamber-specific environmental variation cannot be entirely excluded. Future studies using replicated chambers per treatment would further strengthen inference regarding temperature effects.

In this study, plants were exposed to daily short-term temperature pulses (2 h per day) rather than continuous thermal stress. Therefore, the observed metabolite changes likely reflect metabolic responses to repeated temperature exposure rather than long-term metabolic acclimation.

## 4. Materials and Methods

### 4.1. Experimental Design

A completely randomized factorial design (2 × 3) was used, consisting of two glycine betaine (GB) treatments control without application (GB0) and GB application at the optimal dose (GB1), combined independently with cold- or heat-stress temperature levels. For the cold-stress experiment, the treatments were: control temperature (C0, 25 °C), mild cold stress (C1, 15 °C), and severe cold stress (C2, 5 °C). For the heat-stress experiment, the treatments were: control temperature (H0, 25 °C), mild heat stress (H1, 35 °C), and severe heat stress (H2, 45 °C). Each experiment followed the same factorial structure (2 GB levels × 3 temperature levels), with five replications per treatment combination, totaling 30 experimental units. Statistical analyses were performed using InfoStat 2020d software, and mean comparisons were conducted using Tukey’s test at the 5% significance level. Chambers were set to the three target temperatures for each experiment concurrently. Temperature treatments were applied in independent chambers operated simultaneously under identical environmental control settings to minimize temporal variation among treatments.

Prior to the main experiment, preliminary screening trials were conducted to determine an appropriate glycine betaine (GB) concentration and application frequency for *Passiflora edulis* f. *flavicarpa* under drought conditions. Eight GB concentrations (0, 2, 5, 10, 20, 50, 100, and 200 mM) were evaluated in 3-month-old seedlings subjected to simulated drought stress. Concentrations were assessed primarily based on visible stress symptoms and SPAD chlorophyll readings to determine mitigation efficiency and detect potential phytotoxicity. Lower concentrations (≤50 mM) showed limited visible stress mitigation, whereas 200 mM induced mild chlorosis. The 100 mM treatment consistently maintained SPAD values and reduced visible stress symptoms without apparent toxicity and was therefore selected for use in the main temperature-stress experiment.

### 4.2. Experiment Management

Seeds of *Passiflora edulis* f. *flavicarpa* were sown in germination trays and transplanted to individual pots according to seedling size and phenological development. Plants were grown in containers filled with commercial akadama soil, a granular volcanic clay substrate characterized by slightly acidic pH (5.5–6.5), low electrical conductivity (<0.5 dS m^−1^), total porosity of approximately 50–65%, and moderate water-holding capacity (30–45% *v*/*v*). The substrate contains minimal organic matter (<5%) and very low intrinsic nutrient levels (total nitrogen typically <0.1%), allowing precise control of mineral nutrition. All plants were cultivated using the same substrate batch to ensure uniform growth conditions across treatments. Nutrient management consisted of foliar fertilization with a mixture of 8 g of OAT House Fertilizer No. 1 and 6 g of OAT House Fertilizer No. 2, applied three times per week beginning one month after seedling establishment.

Plants were maintained under uniform growth conditions until they reached three months of age, at which point the experimental period commenced. This age was selected based on the height limitations of the cultivation chambers and to ensure that all seedlings had fully developed functional leaves for metabolite analysis.

Plants were maintained in a cultivation chamber set to 25 °C and approximately 70% relative humidity (RH) under a 12 h light/12 h dark photoperiod throughout the experimental period. Temperature stress treatments were imposed using independent growth chambers set to one of the target temperatures (5, 15, 25, 35, or 45 °C). To apply temperature stress, seedlings were transferred daily to the respective temperature chambers for a 2 h exposure period without light (programmed to be dark time in the main cultivation chamber), after which they were returned to the cultivation chamber under the standard growth conditions described above. This procedure was repeated throughout the 45-day experimental period, thereby simulating recurrent short-duration temperature stress events rather than continuous exposure.

Glycine betaine (GB, 100 mM) was applied via foliar spraying at the onset of the experiment and subsequently every 10 days. This application schedule was selected to maintain consistent GB presence during the experimental period and to examine its association with metabolic responses under contrasting temperature conditions.

To minimize potential variability associated with temperature-dependent growth rates, sampling was standardized by collecting fully expanded leaves of comparable developmental stage across treatments, and metabolite concentrations were expressed on a dry-weight basis.

### 4.3. Experiment Measurement

Fresh leaf samples were collected from each treatment and transported to the Tropical Horticultural Science Laboratory, Tokyo University of Agriculture, for metabolite analysis. Samples were first freeze-dried using a vacuum lyophilizer (EYELA FDM-100, Tokyo Rikakikai Co. Ltd., Tokyo, Japan) for 3 days and subsequently ground. Approximately 100 mg of dried tissue was weighed into 2 mL tubes, each containing a single metal bead. Methanol (250 µL) was added to each sample, followed by homogenization using a shaker (RETSCH MM400, Retsch GmbH, Haan, Germany) for 2 min at 27 Hz. After centrifugation (NICHIRYO C1008-B, Nichiryo Co., Ltd., Tokyo, Japan), 250 µL of chloroform was added, and samples were incubated in a thermomixer (Eppendorf Thermomixer F2.0, Eppendorf AG, Hamburg, Germany) at 37 °C for 3 min at 1200 rpm.

Subsequently, 50 µL of ribitol (internal standard) and 175 µL of ultrapure water were added, and samples were vortexed thoroughly (Vortex Genie 2, Scientific Industries, Inc., Bohemia, NY, USA). The mixtures were then centrifuged (TOMY MX-307, Tomy Seiko Co., Ltd., Tokyo, Japan) at 25 °C, and the supernatant was collected and stored at −80 °C (SANYO MDF-C8V, Sanyo Electric Co., Ltd., Osaka, Japan) until further analysis.

For derivatization, samples were thawed at room temperature, and 80 µL of extract was transferred to new 1.5 mL tubes. Samples were dried using a centrifugal evaporator (EYELA CVE-3110, Tokyo Rikakikai Co. Ltd.) for 2 h, followed by overnight lyophilization at −40 °C. After drying, 40 µL of methoxyamine hydrochloride solution (20 mg mL⁻¹ in pyridine) was added, and samples were incubated at 37 °C for 90 min at 1200 rpm. Subsequently, 50 µL of MSTFA (N-methyl-N-trimethylsilyltrifluoroacetamide) was added, followed by centrifugation and incubation at 37 °C for 30 min at 1200 rpm.

After derivatization, 50 µL of each sample was transferred to GC–MS vials for analysis. Metabolite profiling was performed using a gas chromatograph–mass spectrometer (GC-2010, Shimadzu Corporation, Kyoto, Japan). A total of 50 metabolites were detected, of which nine key metabolites were selected for detailed analysis. Multivariate analysis was conducted using principal component analysis (PCA) in XLSTAT 2025.. The analyzed metabolites included 45 metabolites for heat stress and 47 metabolites for cold stress, including amino acids, organic acids, sugars and related compounds. Because metabolomic datasets contain numerous correlated variables, interpretation focused primarily on overall metabolic patterns revealed by multivariate analysis rather than on isolated changes in individual metabolites. 

To improve visualization and interpretability of metabolite patterns, a subset of representative metabolites was selected for heatmap analysis. Metabolites were chosen based on their biological relevance to stress responses and their contribution to major metabolic pathways, including amino acid metabolism, organic acid metabolism, and carbohydrate metabolism. This targeted selection allowed for clearer identification of treatment-related patterns while avoiding visual overcrowding associated with large datasets. Heatmaps were generated using Z-score-normalized data to highlight relative changes in metabolite abundance across treatments.

To account for multiple hypothesis testing across metabolites, *p*-values were adjusted using the Benjamini–Hochberg false discovery rate (FDR) procedure. The complete metabolite dataset, including raw and FDR-adjusted *p*-values for all detected metabolites, is provided in Supplementary Tables S1 and S2. Representative GC–MS chromatograms, mass spectra, and peak integration outputs are provided in the Supplementary Materials (Figures S1–S10).

## 5. Conclusions

This study characterizes metabolite responses of yellow passion fruit (*Passiflora edulis* Sims f. *flavicarpa*) seedlings exposed to contrasting temperature stresses and examines how exogenous glycine betaine (GB) application is associated with changes in primary metabolism under these conditions. Heat and cold stress produced distinct metabolite patterns, indicating substantial temperature-dependent shifts in central carbon and nitrogen metabolism. Heat stress was associated with coordinated changes in amino acids, organic acids, sugars, polyamines, and γ-aminobutyric acid, whereas cold stress was characterized primarily by increased accumulation of soluble carbohydrates accompanied by reductions or redistribution of several amino acid and organic acid pools.

Exogenous application of glycine betaine did not eliminate stress responses but was associated with altered metabolite distributions under both thermal extremes. Principal component analysis indicated that GB-treated samples occupied distinct regions of multivariate space relative to untreated stressed plants, suggesting that GB corresponded with modified metabolic configurations rather than restoration of control-like profiles.

Overall, these results provide a metabolomic characterization of temperature-associated metabolic responses in passion fruit seedlings and highlight how glycine betaine treatment is associated with changes in primary metabolism under contrasting thermal conditions. The findings contribute metabolite-level observations that may support future studies integrating metabolomics with physiological and molecular analyses to better understand temperature stress responses in *Passiflora edulis*.

## Figures and Tables

**Table 1:** Effect of GB application on proline, γ-aminobutyric acid (GABA), glutamic acid, glutamine, putrescine, ornithine, valine, sucrose, glucose and malic acid concentration values of seedlings of passion fruit. “H0” refers to control heat, “H1” indicates mild heat, and “H2” denotes severe heat. “GB0” indicates no application of glycine betaine, whereas “GB1” signifies glycine betaine application.

			Metabolite Concentration (μmol/g)			
		Proline	γ-Aminobutyric Acid (GABA)	Glutamic Acid	Glutamine	Putrescine
Treatments	H0GB0	2.39 ± 0.35 c	3.81 ± 0.26 c	0.14 ± 0.006 d	0.004 ± 0.0005 b	0.42 ± 0.01 e
	H0GB1	8.81 ± 0.01 a	3.87 ± 0.01 c	0.20 ± 0.002 d	0.01 ± 0.0002 b	0.85 ± 0.004 b
	H1GB0	3.09 ± 0.04 a	2.19 ± 0.05 e	0.44 ± 0.004 c	0.08 ± 0.004 b	0.39 ± 0.01 f
	H1GB1	4.04 ± 0.15 b	7.60 ± 0.07 a	1.23 ± 0.02 a	0.26 ± 0.04 a	1.29 ± 0.005 a
	H2GB0	2.21 ± 0.04 c	2.87 ± 0.08 d	0.15 ± 0.004 d	0.02 ± 0.0002 b	0.55 ± 0.005 d
	H2GB1	9.12 ± 0.14 d	6.75 ± 0.05 b	0.66 ± 0.004 b	0.01 ± 0.001 b	0.67 ± 0.005 c
		**Ornithine**	**Valine**	**Sucrose**	**Glucose**	**Malic Acid**
	H0GB0	0.07 ± 0.002 c	0.06 ± 0.007 d	6.66 ± 0.07 d	8.97 ± 0.04 d	14.02 ± 0.66 d
	H0GB1	0.17 ± 0.0002 c	0.82 ± 0.002 a	9.23 ± 0.02 d	13.24 ± 0.08 b	16.14 ± 0.78 d
	H1GB0	1.30 ± 0.01 b	0.06 ± 0.003 d	48.96 ± 0.53 a	2.38 ± 0.15 f	27.93 ± 0.48 b
	H1GB1	2.39 ± 0.09 a	0.31 ± 0.007 c	34.28 ± 2.62 b	11.95 ± 0.21 c	33.80 ± 0.31 a
	H2GB0	0.19 ± 0.004 c	0.03 ± 0.005 e	5.83 ± 0.15 d	6.26 ± 0.13 e	14.28 ± 0.39 d
	H2GB1	0.21 ± 0.002 c	0.36 ± 0.004 b	16.58 ± 0.38 c	15 ± 0.13 a	20.39 ± 0.1 c

Different letters mean different significant results at Tukey at 5%.

**Table 2:** Effect of GB application on proline, sucrose, glucose, fructose, sorbitol, inositol, malic acid, citric acid, threonine and putrescine concentration values of seedlings of passion fruit. “C0” refers to control cold, “C1” indicates mild cold, and “C2” denotes severe cold. “GB0” indicates no application of glycine betaine, whereas “GB1” signifies glycine betaine application.

			Metabolite Concentration (μmol/g)			
		Proline	Sucrose	Glucose	Fructose	Sorbitol
Treatments	C0GB0	10.02 ± 0.23 a	1.06 ± 0.01 c	0.03 ± 0.0008 b	8.61 ± 0.06 a	14.29 ± 0.18 a
	C0GB1	10.28 ± 0.21 a	1.07 ± 0.02 c	0.03 ± 0.0007 b	8.41 ± 0.11 a	13.67 ± 1.16 a
	C1GB0	0.04 ± 0.004 b	2.72 ± 0.07 b	1.50 ± 0.22 b	0.18 ± 0.06 b	0.26 ± 0.006 b
	C1GB1	0.08 ± 0.02 b	7.19 ± 0.91 a	3.05 ± 0.55 a	0.19 ± 0.01 b	0.14 ± 0.007 b
	C2GB0	0.05 ± 0.008 b	4.02 ± 0.3 b	3.32 ± 0.87 a	0.37 ± 0.14 b	0.19 ± 0.009 b
	C2GB1	0.25 ± 0.08 b	8.93 ± 0.66 a	3.14 ± 0.8 a	0.27 ± 0.02 b	0.22 ± 0.007 b
		**Inositol**	**Malic Acid**	**Citric Acid**	**Threonine**	**Putrescine**
	C0GB0	11.21 ± 0.13 a	27.30 ± 0.13 a	6.18 ± 0.05 a	0.03 ± 0.001 a	0.97 ± 0.009 a
	C0GB1	11.62 ± 0.54 a	27.81 ± 0.24 a	6.21 ± 0.05 a	0.03 ± 0.0008 a	0.94 ± 0.01 a
	C1GB0	0.53 ± 0.01 b	1.60 ± 0.23 c	1.12 ± 0.08 b	0.002 ± 0.0002 c	0.27 ± 0.008 c
	C1GB1	0.67 ± 0.04 b	4.17 ± 0.66 b	2.17 ± 0.19 b	0.01 ± 0.0006 b	0.34 ± 0.02 b
	C2GB0	4.50 ± 0.05 b	2.59 ± 0.52 c	0.81 ± 0.03 d	0.001 ± 0.0003 c	0.18 ± 0.006 d
	C2GB1	0.59 ± 0.1 b	5.58 ± 1.76 b	1.93 ± 0.28 b	0.01 ± 0.0005 b	0.38 ± 0.03 b

Different letters mean different significant results at Tukey at 5%.

## Data Availability

The raw data supporting the conclusions of this article will be made available by the authors on request.

## Supplementary Materials

The following supporting information can be downloaded at: https://www.mdpi.com/article/10.3390/ijms27093811/s1.
