# Supplementary material for: Glycine Betaine-Induced Metabolic Responses Under Heat and Cold Stress in Passiflora edulis f. flavicarpa
Source: Int J Mol Sci. 2026 Apr 24;27(9):3811. doi: 10.3390/ijms27093811 (PMC13163230; doi:10.3390/ijms27093811)
Supplement: Supplementary file 1 [file ijms-27-03811-s001.zip › Supplementary Figures.pdf]

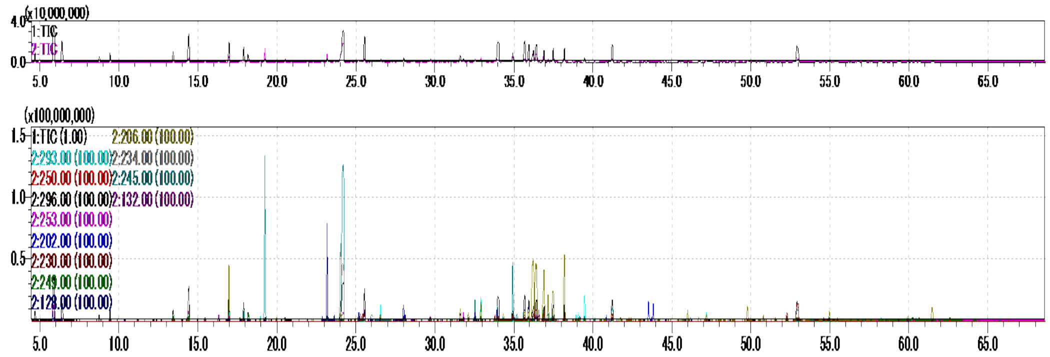

Supplementary Figure S1. Representative GC–MS chromatograms of metabolite extracts under heat stress conditions showing peak separation.

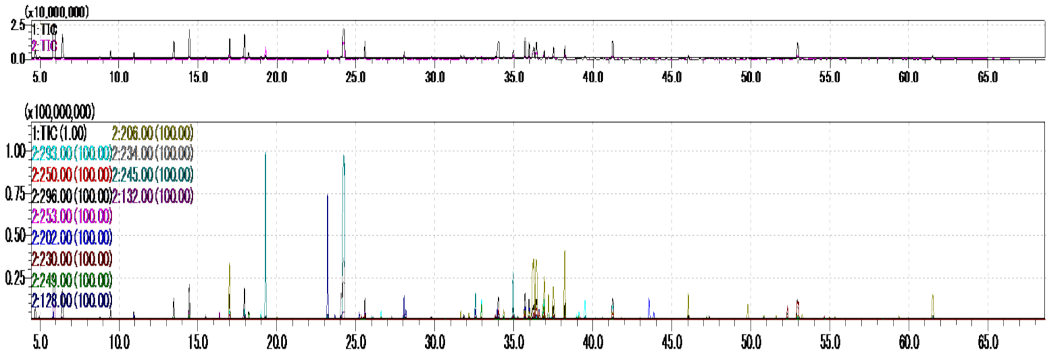

Supplementary Figure S2. Representative GC–MS chromatograms of metabolite extracts under cold stress conditions showing peak separation.

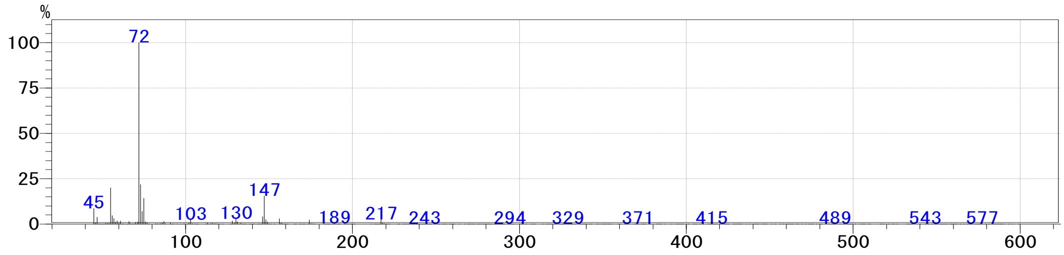

Supplementary Figure S3. Representative mass spectra used for metabolite identification in H0GB01 sample (pyruvic acid).

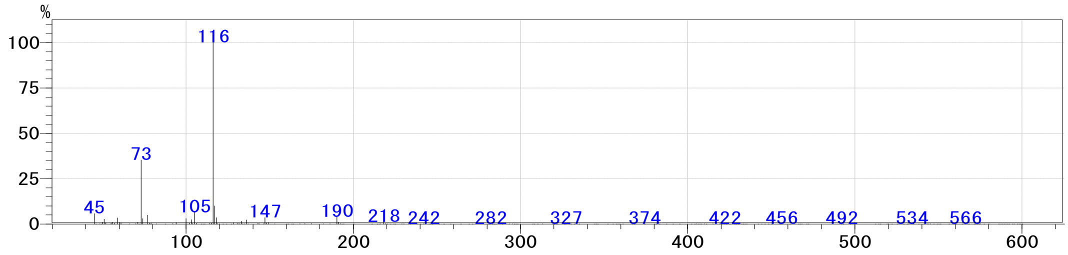

Supplementary Figure S4. Representative mass spectra used for metabolite identification in H0GB01 sample (alanine).

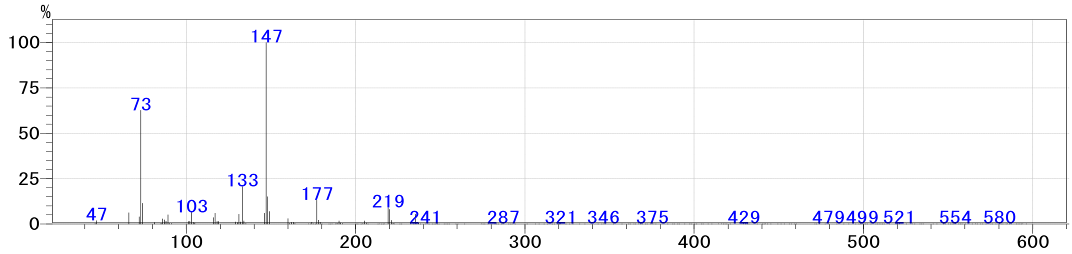

Supplementary Figure S5. Representative mass spectra used for metabolite identification in H0GB01 sample (oxalic acid).

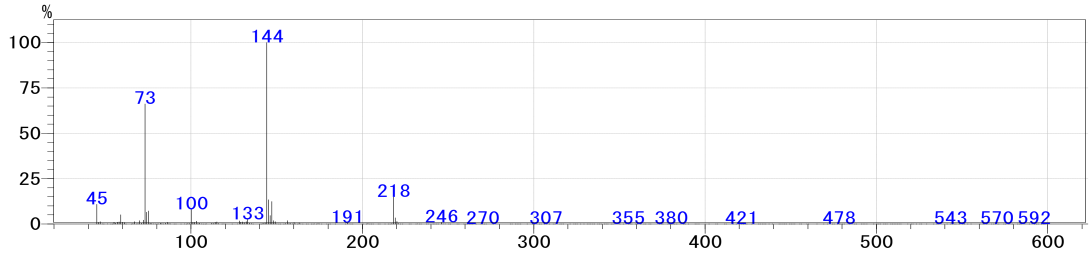

Supplementary Figure S6. Representative mass spectra used for metabolite identification in H0GB01 sample (valine).

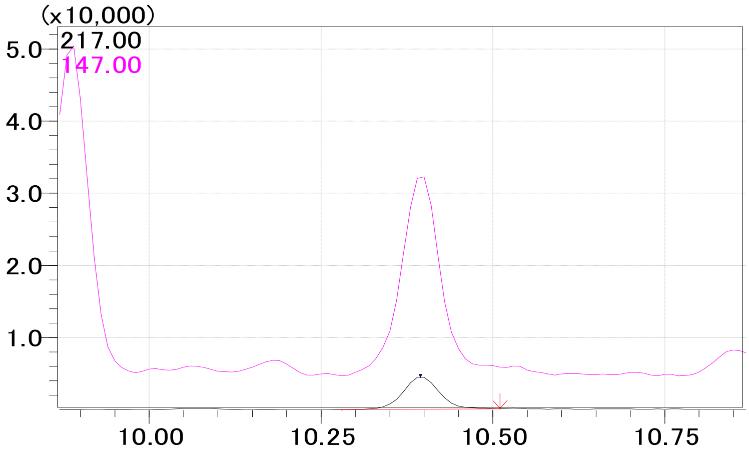

Supplementary Figure S7. Example of peak integration used for metabolite quantification (pyruvic acid).

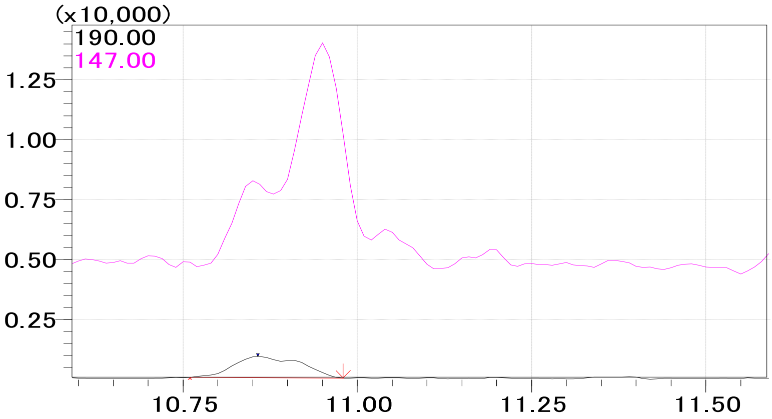

Supplementary Figure S8. Example of peak integration used for metabolite quantification (alanine).

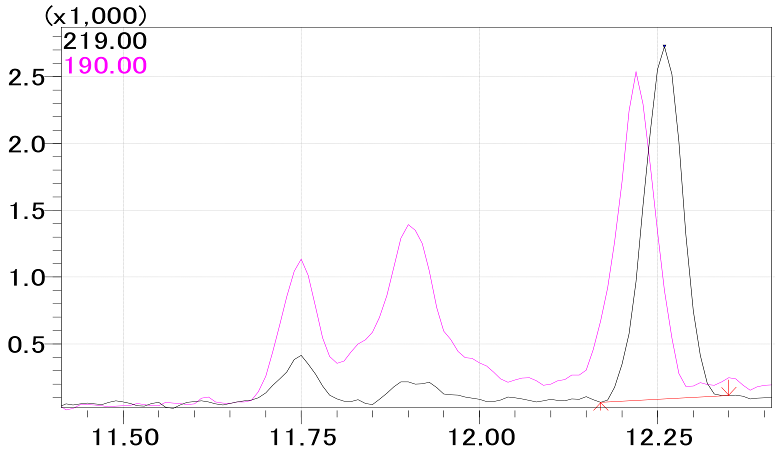

Supplementary Figure S9. Example of peak integration used for metabolite quantification (oxalic acid).

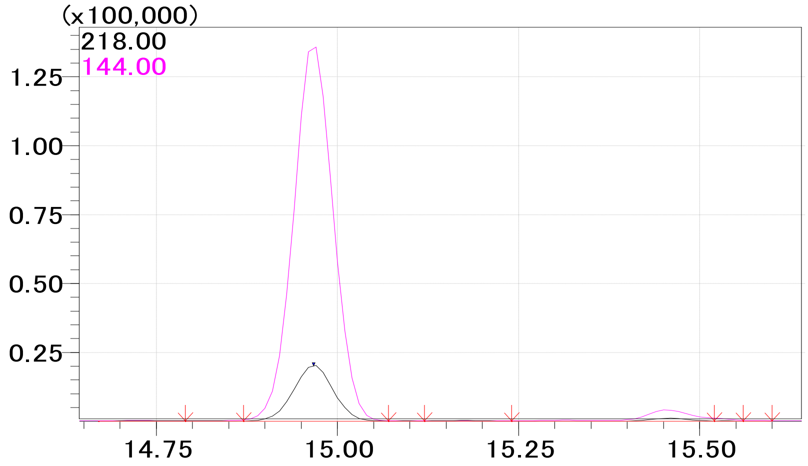

Supplementary Figure S10. Example of peak integration used for metabolite quantification (valine).
